# Awareness for artifacts in fluorescence microscopy of β-TCP

**DOI:** 10.1186/s13104-024-06781-0

**Published:** 2024-04-29

**Authors:** Marco Waldmann, Marc Bohner, Anna Baghnavi, Bianca Riedel, Michael Seidenstuecker

**Affiliations:** 1 G.E.R.N. Tissue Replacement, Regeneration & Neogenesis, Department of Orthopedics and Trauma Surgery, Faculty of Medicine, Medical Center-Albert-Ludwigs-University of Freiburg, Albert-Ludwigs- University of Freiburg, Hugstetter Straße 55, 79106 Freiburg, Germany; 2 Robert Mathys Foundation RMS, Bischmattstr. 12, Bettlach, 2544 Switzerland

**Keywords:** β-TCP, Ceramic, Fluorescence, Immunofluorescence, Live/Dead, Artifacts, Technovit

## Abstract

Fluorescence analysis of β-TCP ceramics is often used to describe cells found on said ceramics. However, we found, to our knowledge, so far undescribed artifacts which might sometimes be hard to differentiate from cells due to shape and fluorescence behavior. We tried prolonged ultrasound washing as well as Technovit 9100 fixation to reduce these artifacts. While untreated dowels showed no reduction in artifacts no matter the further treatment, Technovit fixation reduced the artifacts with even further reduction achieved by mechanical cleaning. As a consequence, scientists working with these dowels and likely even other types should try to avoid creating false positive results by considering the existence of these artifacts, checking additional filters for unusual fluorescence and by reducing them by using Technovit fixation when possible.

## Introduction

β-TCP ceramics are frequently used in orthopedic settings and well researched [1–6]. They are biodegradable ceramics which hold osteoinductive and osteoconductive properties and are able to form a strong bond with host tissue. The mechanical properties of β-TCP however are limited. They can be seen as an alternative to the “gold standard” of autologous bone grafts, which, although they have high osteoinductive and osteogenic capacities, come with disadvantages like an increased risk of infection due to a second surgical site, limitation of available graft material and likewise limited mechanical properties, making them a great choice for smaller but not for large-scale bone defects. In situations where these disadvantages outweigh the advantages of autologous bone grafts, alternatives like β-TCP come into play [7–9]. 

Research of β-TCP includes analysis of surfaces using fluorescence microscopy as one common technique to characterize cells found on these surfaces. For example, live/dead assays to access viability of cells [10], immunofluorescence [10] or proof of loading using FITC conjugated drugs [11] are common practice. Nonetheless, fluorescence microscopy can also lead to unwanted effects or artifacts, possibly resulting in misinterpretation. The process of photobleaching describes the loss of fluorescence of a fluorochrome molecule due to chemical destruction after exposure to excitation light [12]. Consequently, originally positive signals might be missed if the excitation time is too long. Techniques to examine specific proteins like immunolabeling also rely on the specificity of the primary antibody in use [13]. Without proper testing, unintended proteins might also produce positive signals. With immunolabeling, incomplete protein tagging can also occur if the targeted cells are not be properly permeabilized [13].

Autofluorescence describes the inherent fluorescence without addition of an exogenous fluorescent agent found in some substances [14]. This phenomenon can be found in various instances. Examples include certain plastics [15], plants [16] and also human tissue [17, 18]. Depending on the signal strength, this phenomenon could also be falsely interpreted as a fluorescence caused by addition of external fluorescent molecules, if not properly accounted for in the first place. Another problem might occur, when different fluorescent agents with overlapping wavelength ranges of emitted light are chosen and not properly separated with adequate filters [19].

During our analysis of β-TCP, we noticed artifacts that, to our knowledge, have not yet been addressed and could potentially be confused with cells during fluorescence microscopy in an appropriate context.

## Material

**Table:** 

Material	Information
Ethanol 99%	SAV Liquid Production GmBH, Flintsbach am Inn, Germany
Technovit 9100 Combipack (contains: 1 × 1000 ml basic solution 1 × 120 g PMMA powder 8 × 1 g hardener 1 1 × 10 ml hardener 2 1 × 5 ml regulator), #64,715,444	Kulzer GmbH, Hanau, Germany
Cerasorb M Cylinder (Lot D147.501043)	Curasan, Kleinostheim, Germany
Kimtech® Science Precision Wipes, #7552	Irving, Tx, USA
Filter: Alizarin/Xylenolorange; Calcein; Cy5&AF647; Tetracyclin; FITC/Cy5 H Dualband Filter	AHF, Tuebingen, Germany
Olympus BX51 fluorescent microscope, equipped with a 10x objective	Olympus, Tokyo, Japan
UV-Lightsource X-Cite Series 120 Q	Excelitas Technologies, Waltham, USA

## Methods

**Fig. 1:**
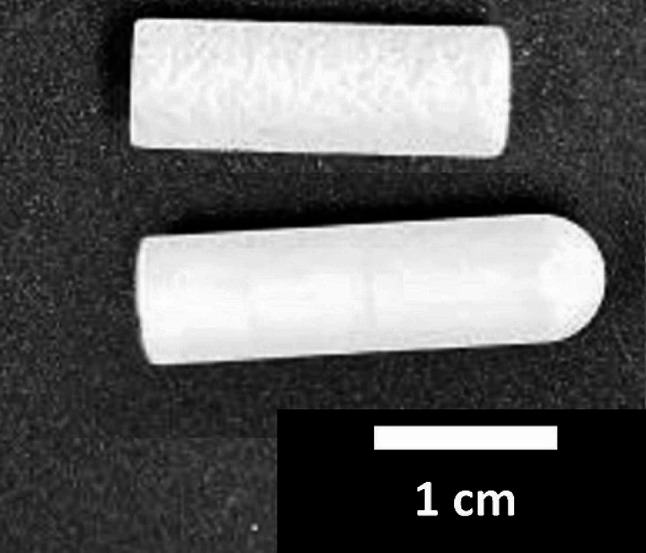
Picture of the two types of dowels analyzed within this work. The upper dowel was manufactured by Curasan, the lower dowel was produced by the RMS. A scalebar in cm is provided within the picture

We analyzed the surface of microporous β-TCP dowels (Ø 7 mm x L 26 mm, median pore diameter 5 μm, 40% total porosity [20], Fig. 1) produced by the RMS according to our specifications [21, 22] using fluorescence microscopy. To reduce the artifacts we tried different procedures of either ultrasound bathing dowels in 70% ethanol followed by distilled water for 10 min each (frequency 80/50 Hz, 26 °C) or ultrasound bathing in 70% ethanol followed by distilled water for 2 h with the same parameters. Sucking 5 mL of distilled water through the ceramics by applying a slight vacuum (650 mBar) to a flow chamber [23, 24] was tested as another method of reducing the artifacts. After ultrasound bathing for 10 min each, some dowels were fixated using Technovit 9100 new. Mechanical cleaning using cellulose wipes was tried in addition for Technovit fixated dowels and dowels ultrasound bathed for 10 min each. Untreated dowels (Ø 7 mm x L 20 mm, median pore diameter 37 μm, 62% total porosity [20], Fig. 1) purchased from a different manufacturer, Curasan (Cersasorb M, Fig. 1), were also analyzed.

Images of the dowels were taken at a defined position using an Olympus BX51 fluorescent microscope equipped with a 10x objective (Olympus, Tokyo, Japan) and filters for five different wavelengths. Artifact counts were determined for the whole image using ImageJ automated particle counting for images captured with a FITC/Cy5 H Dualband filter. All data are presented as mean ± standard deviation.

## Results

**Fig. 2:**
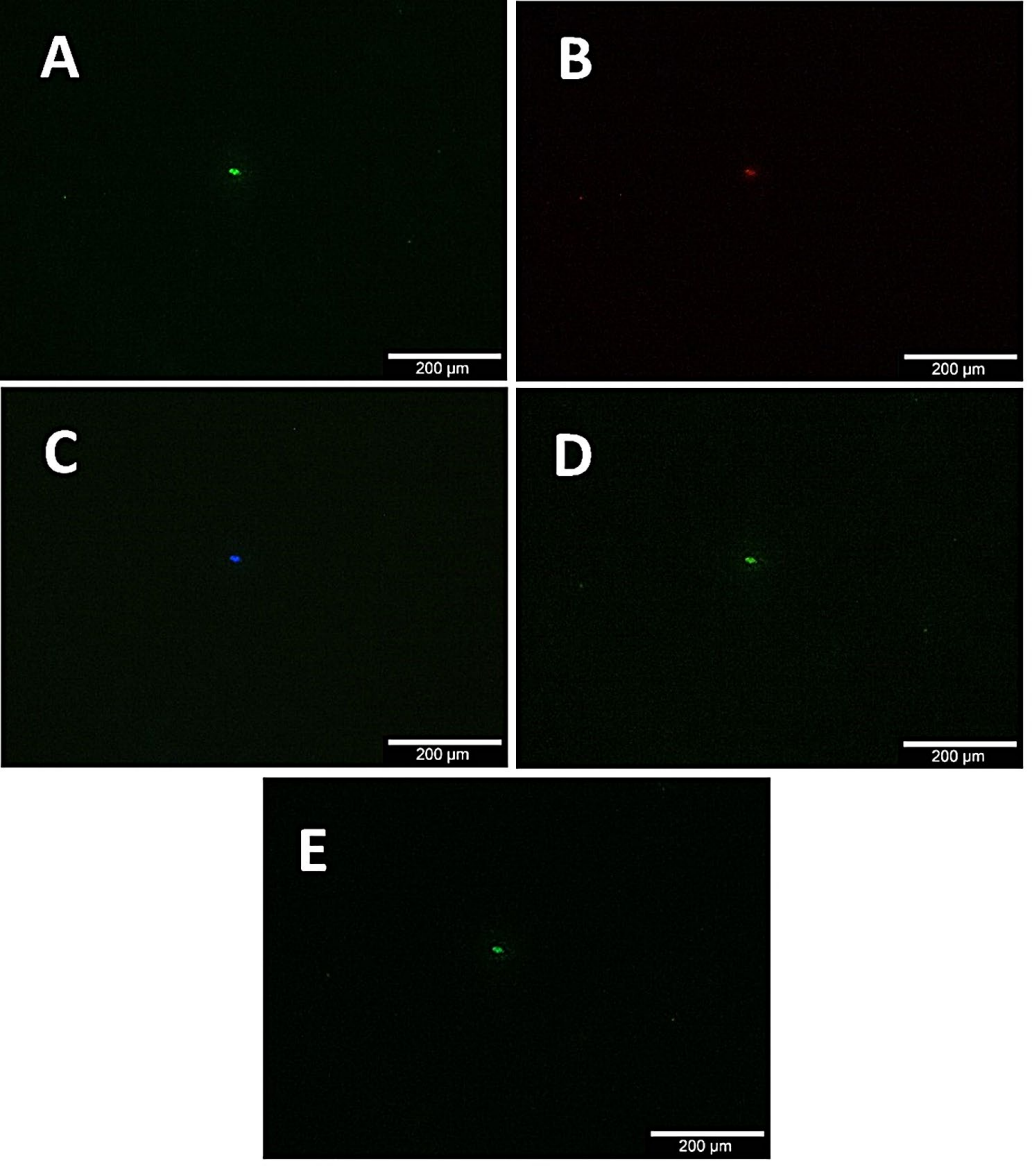
Exemplary picture of fluorescence artifacts found on untreated, microporous β-TCP dowels provided by the RMS. All Frames show the same area using different excitation filters for Alizarin/Xylenolorange (**Frame A**), Tetracyclin (**Frame B**), Cy5&AF647 (**Frame C**), FITC/Cy5 H Dualband Filter (**Frame D**) and Calcein (**Frame E**). The Pictures were taken with an Olympus BX51 fluorescent microscope equipped with a 10x objective (Olympus, Tokyo, Japan)

The artifacts varied in size and shape but were often found to be round and had diameters ranging from approximately 5 to 80 μm. The artifacts were found on every dowel regardless of procedure and were fluorescent for all five filters analyzed (Fig. 2). Completely untreated (8.67 ± 0.58), ultrasound bathed for 10 min each (12 ± 7.21) as well as dowels ultrasound bathed for 2 h each (8.67 ± 5.03) showed said shapes for all wavelengths analyzed with no reduction in any group. Dowels fixated in Technovit showed a reduced number of artifacts (3.67 ± 0.58) compared to untreated dowels. For Technovit fixated dowels, mechanical cleaning reduced the artifacts even further (1.33 ± 0.58) but despite all efforts never achieved complete removal. Mechanical cleaning showed no effect for dowels ultrasound bathed for 10 min each (12.00 ± 1.73). (Table 1)

Untreated dowels by Curasan, were found to show said artifacts with slightly lower numbers than RMS dowels (5.33 ± 1.15). Cleaning the ceramics using 5 mL of distilled water under a slight vacuum was unsuccessful in reducing the artifacts (7.33 ± 2.31). (Table 1)

**Table 1:** Result of ImageJ counting for images captured with a FITC/Cy5 H Dualband filter of each analyzed group (*n* = 3) presented as mean ± standard deviation

Analyzed dowels		Artifacts
Curasan	uncleaned	5.33 ± 1.15
RMS	uncleaned	8.67 ± 0.58
	10 min ultrasound bath	12 ± 7.21
	10 min ultrasound bath, mechanically cleaned	12 ± 1.73
	2 h ultrasound bath	8.67 ± 5.03
	slight vacuum	7.33 ± 2.31
	Technovit	3.67 ± 0.58
	Technovit, mechanically cleaned	1.33 ± 0.58

## Discussion

While the origin of the observed artifacts is unknown to us, impurities during the production process or surface modification during ceramic sintering [25] seem to be a reasonable explanation. The artifacts may be limited to the analyzed type of β-TCP samples produced by RMS and Curasan but for the evaluation of cells on β-TCP by fluorescence microscopy, researchers should be aware of the artifacts described in this note and prevent false positive results by considering their existence, even for other β-TCP products. With no fluorescence filter producing negative results, the artifacts can also be expected to extent to different wavelengths.

In general, in regard of the described artifacts, Technovit fixation followed by cautious mechanical cleaning seems to be the most suitable procedure, if tolerated by the type of sample that has to be evaluated. In addition, and especially when untreated dowels are analyzed, checking a completely untreated dowel for the described unwanted artifacts as a reference, as well as separating artifacts from actual positive results by controlling other wavelengths that would not normally show fluorescence signals with the chosen parameters, seems to be a suitable method for preventing false positive results. Ultrasound bathing probably does not help to reduce this type of artifact.

Another possible option to reduce the effect of autofluorescence is the exploitation of differences in fluorescence lifetimes between autofluorescent substances and fluorescent agents with longer lifetimes [26–28].

Assuming autofluorescence of impurities during production as the source of artifacts and depending on the fluorescent agents in use, trying to find differences between the artifacts and structures labeled with exogenous markers via means of fluorescence lifetime imaging microscopy [29, 30] may prove useful in the future to correctly differentiate these artifacts from cells.

## Limitations

Only dowels produced by the RMS and Curasan, and only from one batch each, were analyzed. Also, the filters were limited to five different types, while we expect the bandwidth of fluorescenting wavelengths to be even broader than observed in this work.

## Data Availability

The datasets used and/or analyzed during the current study are available from the corresponding author on reasonable request.

## Abbreviations

RMS: Robert Mathys Foundation
β-TCP: Beta-tricalcium phosphate
